# Tailoring Alkalized and Oxidized V_2_CT_x_ as Anode Materials for High-Performance Lithium Ion Batteries

**DOI:** 10.3390/ma17143516

**Published:** 2024-07-16

**Authors:** Yuxuan Zhang, Lin Gao, Minglei Cao, Shaohui Li

**Affiliations:** 1 Hubei Key Laboratory of Energy Storage and Power Battery, School of Mathematics, Physics and Optoelectronic Engineering, Hubei University of Automotive Technology, Shiyan 442002, China; 19945067242@163.com (Y.Z.); cml07114052@163.com (M.C.); 2 School of Materials Science and Engineering, Zhengzhou University, Zhengzhou 450001, China

**Keywords:** lithium ion batteries, anode materials, V_2_CT_x_ MXenes, cycling performance, ion transfer kinetics

## Abstract

V_2_CT_x_ MXenes have gained considerable attention in lithium ion batteries (LIBs) owing to their special two-dimensional (2D) construction with large lithium storage capability. However, engineering high-capacity V_2_CT_x_ MXenes is still a great challenge due to the limited interlayer space and poor surface terminations. In view of this, alkalized and oxidized V_2_CT_x_ MXenes (OA-V_2_C) are envisaged. SEM characterization confirms the accordion-like layered morphology of OA-V_2_C. The XPS technique illustrates that undergoing alkalized and oxidized treatment, V_2_CT_X_ MXene replaces -F and -OH with -O groups, which are more conducive to pseudocapacitive properties as well as Na ion diffusion, providing more active sites for ion storage in OA-V_2_C. Accordingly, the electrochemical performance of OA-V_2_C as anode materials for LIBs is evaluated in this work, showing excellent performance with high reversible capacity (601 mAh g^−1^ at 0.2 A g^−1^ over 500 cycles), competitive rate performance (222.2 mAh g^−1^ and 152.8 mAh g^−1^ at 2 A g^−1^ and 5 A g^−1^), as well as durable long-term cycling property (252 mAh g^−1^ at 5 A g^−1^ undergoing 5000 cycles). It is noted that the intercalation of Na^+^ ions and oxidation co-modification greatly reduces F surface termination and concurrently increases interlayer spacing in OA-V_2_C, significantly expediting ion/electron transportation and providing an efficient way to maximize the performance of MXenes in LIBs. This innovative refinement methodology paves the way for building high-performance V_2_CT_x_ MXenes anode materials in LIBs.

## 1. Introduction

Currently, lithium ion batteries (LIBs) are undoubtedly considered the main energy storage option for portable electronics, electromotives and stationary smart grids. As we all know, graphite is regarded as the common anode material in LIBs because of its low cost and satisfactory voltage plateau. However, the limited capacity severely impedes the energy density of the whole battery. Therefore, exploiting high-capacity anode materials with steady electrochemical behavior is beneficial to realizing high-energy-density LIBs. Transition metal carbides and nitrides (MXenes), as a family of 2D layered structures, have been fully investigated since 2011 [1,2]. MAX and M_n+1_AX_n_ phases indicate hexagonal layered transition metal carbides and nitrides. Commonly, MXenes can be obtained via etching the A-element from the layered MAX phase [3,4,5]. The obtained layered MXenes have a general chemical formula, which can be denoted as M_n+1_X_n_T_x_, where M is a transition metal element (Ti, V, etc.), X is a carbon or nitrogen element, n = 1, 2 or 3, and T_x_ stands for adsorbed functional groups at the surface such as -F, -O and -OH [6,7,8]. MXenes generally possess high electronic conductivity, excellent mechanical behavior, high surface area and large interlayer spacing, as well as rich surface functional groups [9,10,11,12,13,14]. In this regard, MXenes show great application prospects in rechargeable batteries [15], supercapacitors [16], photocatalysts [17], catalysts [18] and other fields. Among the MXenes family, most of the exploitations focus on Ti_3_C_2_T_x_, mainly because of its mature preparation and stripping method [19,20,21,22]. Compared with Ti_3_C_2_T_x_, V_2_CT_x_ not only occupies better conductivity but also shows higher capacity and lower ion transport barrier [23]. As the anode material in LIBs, the theoretical capacity of V_2_CT_x_ can reach as high as 940 mAh g^−1^, which is much higher than that of other MXenes [24]. However, it is still a huge challenge to completely remove the functional groups such as -F and -OH of the V_2_CT_x_, which largely hinders the transmission of Li^+^ ions and reduces its capacity [25,26,27,28,29,30].

In order to ameliorate the electrochemical performance of V_2_CT_x_, it is necessary to reasonably enhance its reaction kinetics and ion transport efficiency. For example, Wang et al. reported the intercalation of Co^2+^ into hydrated bilayers of V_2_C MXene to expand its interlayer spacing and form a stable V-O-Co bond between the layers, which considerably improves the performance of V_2_C MXene with a high specific capacity of 1117.3 mAh g^−1^ [31]. Similarly, Sn^4+^-decorated V_2_C MXene demonstrated improved performance, as well as exceptional rate and cyclic stability in LIBs due to the expanded interlayer spacing and the creation of V-O-Sn bonds. Recently, a co-modification of V_2_CT_X_ MXene with K^+^ ions and -O functional groups was proposed. Notably, K^+^ ions were introduced to stabilize the interlayer structure and prevent nanosheet aggregation, while the -O terminal group was selectively added to the MXene surface to enhance the reversible capacity for Li^+^ storage. The co-modified V_2_CT_X_ MXene exhibits superior reversible specific capacities of 671.8 mA h g^−1^ at 0.1 A g^−1^ and 318 mA h g^−1^ at 1.0 A g^−1^ [32]. Additionally, Ca^2+^ ions were introduced into the interlayer of V_2_CT_X_ MXenes through alkalization and ion-exchange processes. Structural and chemical analyses revealed modifications in surface terminations and interlayer spacing. Calcium ion intercalation resulted in a larger interlayer distance, facilitating efficient Li^+^ transport channels and providing more space for ion storage. Furthermore, the ion-exchange process reduced F surface terminations, enhancing electron conductivity and activating the surface terminations of V_2_CT_X_ MXenes for participation in redox reactions. Thus, adjusting the layer structure of V_2_CT_X_ via cation intercalation and control techniques significantly promotes ion diffusion kinetics and boosts surface electrochemical reaction activity, leading to superior electrochemical performance [33,34,35,36,37]. 

Inspired by the previous works, here, the alkalized and oxidized V_2_CT_x_ (OA-V_2_C) was prepared based on a hydrothermal reaction. Abundant O-terminal groups on the surface after oxidation effectively improve the diffusion kinetics and structural stability of Li^+^. The surface terminations, such as hydroxyl (-OH), oxygen (-O), or fluorine (-F), can significantly increase the electrochemical activity. These functional groups could supply additional sites for Li ion storage, leading to higher capacity. Concurrently, the Na^+^ intercalation effectively enlarges the interlayer of V_2_CT_x_. Therefore, the OA-V_2_C electrode delivers enhanced capacity and excellent electrochemical performance, showing excellent performance with high reversible capacity (601.0 mAh g^−1^ at 0.2 A g^−1^ over 500 cycles) and competitive rate performance (222.2 mAh g^−1^ and 152.8 mAh g^−1^ at 2 A g^−1^ and 5 A g^−1^) as well as durable long-term cycling property (252 mAh g^−1^ at 5 A g^−1^ undergoing 5000 cycles).

## 2. Materials and Methods

### 2.1. Material Preparation 

Multilayered V_2_CT_x_ (M-V_2_C) was obtained based on a modified etching method [1,2]. In order to obtain M-V_2_C, 1 g NaF powders purchased from Sinopharm Chemical Reagent Co., Ltd (Shanghai, China) were added into a 100 mL glass beaker, followed by adding 10 mL HCl (30%) purchased from Sinopharm Chemical Reagent Co., Ltd (Shanghai, China) and deionized water with a volume ratio of 1:1. Then, 1 g V_2_AlC (Nanjing XFNANO Materials Tech Co., Ltd, Nanjing, China) powders were slowly added to the above mixed solution and stirred for 30 min. Subsequently, the mixture was transferred into a 100 mL Teflon-lined stainless-steel autoclave and heated at 90 °C for 7 days. The resulting solution was centrifuged and washed repeatedly until the pH ≈ 7 and then freeze-dried to acquire M-V_2_C. The oxidized V_2_C MXene (O-V_2_C) was fabricated by the hydrothermal method. Specifically, 0.2 mL H_2_O_2_ (30%) was mixed with 30 mL deionized water under stirring for 5 min at room temperature. Then, the prepared M-V_2_C (0.2 g) was added to the mixture and stirred for 30 min. The obtained solution was then transferred into a Teflon-lined stainless-steel autoclave and heated at 140 °C for 24 h. Subsequently, the resultant product was washed with deionized water and alcohol several times to acquire O-V_2_C. An amount of 0.2 g M-V_2_C was added into 1 M NaOH solution (30 mL) and stirred for 30 min at room temperature. Subsequently, the acquired solution was transferred into a Teflon-lined stainless-steel autoclave and underwent a hydrothermal process at 140 °C for 24 h. Then, the obtained black sample was washed with deionized water and alcohol several times to acquire alkalized V_2_C MXene (A-V_2_C). The prepared M-V_2_C (0.2 g) was added into 1 M NaOH solution (30 mL) with 0.2 mL H_2_O_2_ and stirred for 30 min at room temperature. Then, it was transferred into a Teflon-lined stainless-steel autoclave and heated at 140 °C for 24 h. The product was washed with deionized water and alcohol several times, then dried in the oven at 60 °C for 12 h to obtain the oxidized and alkalized V_2_C MXene (OA-V_2_C). 

### 2.2. Material Characterizations

To determine the external morphologies of the samples, scanning electron microscopy (SEM, JEOL, Tokyo, Japan, JSM-7500F) was performed. X-ray diffraction spectra were acquired via the JEOL-Smart Lab facility. Chemical states were identified by X-ray photoelectron spectroscopy (XPS, Shimadu, AXIS Supra, Kyoto, Japan). An aluminum Kα X-ray source delivering a photon energy of 1486.6 eV served as the excitation mechanism. XPS PEAK4.1 software was used to fit the XPS date employing the C 1s peak at 284.8 eV for adventitious carbon as a reference. The Shirley background subtraction method was employed to fit the XPS data.

### 2.3. Electrochemical Characterizations 

The electrochemical properties were evaluated using CR2032 coin half cells. Working electrodes were prepared from a mixture consisting of 70 wt% active materials, 20 wt% acetylene black and 10 wt% polyvinylidene fluoride (PVDF) dissolved in N-methyl-2-pyrrolidone (NMP). This slurry was then coated onto copper foil and dried in a vacuum at 60 °C for 16 h. The electrode mass loading was 2 mg cm^−2^. Lithium foil served as the counter electrode, and glass fiber acted as the separator. The electrolyte used was 1 M LiPF_6_ in a solution of ethylene carbonate (EC) and dimethyl carbonate (DEC) in a 1:1 volume ratio, with 5% fluoroethylene carbonate (FEC) added. The battery assembly took place in an argon-filled glove box, maintaining H_2_O and O_2_ concentrations below 0.01 ppm. A battery testing system (CT2001, LANHE, Guangzhou, China) assessed the cycling and rate performance within a voltage range of 0.01 to 3.0 V. Cyclic voltammetry (CV) was performed using a CHI 760E electrochemical workstation between 0.01 and 3.0 V. Electrochemical impedance spectroscopy (EIS) was also carried out using the same workstation, spanning from 100 kHz to 0.01 Hz. 

## 3. Results and Discussion

**Figure 1:**
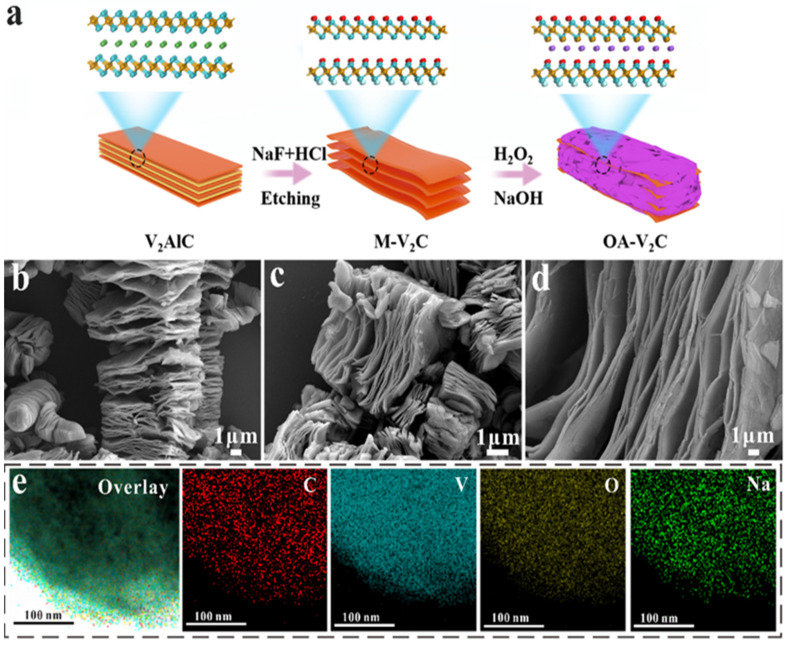
(**a**) Schematic illustration of the preparation process of OA-V_2_C. SEM images of (**b**) M-V_2_C and (**c**,**d**) OA-V_2_C. (**e**) Elemental mappings of OA-V_2_C.

**Figure 2:**
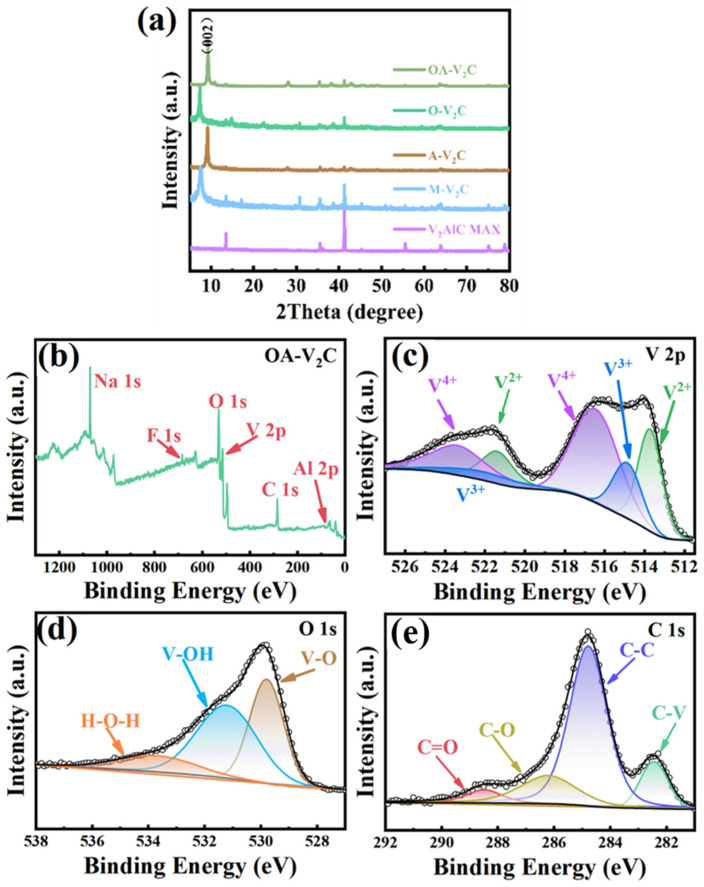
(**a**) XRD pattern of the four samples. (**b**) The survey XPS spectrum of OA-V_2_C. The corresponding high-resolution spectra of (**c**) V 2p, (**d**) O 1s and (**e**) C 1s.

The synthesis process and structure of OA-V_2_C are schematically shown in Figure 1a. It needs to be mentioned that the V_2_AlC MAX displayed a typical dense microstructure (Figure S1a), while the accordion shape of V_2_C MXenes was obtained by selectively etching the Al layers in the V_2_AlC MAX precursor via the mixed solution of HCl and NaF (Figure S1b,c). Afterwards, the obtained multi-layered V_2_C MXenes (M-V_2_C) were added into the mixed solution of NaOH and H_2_O_2_ for a hydrothermal reaction to acquire Na^+^ intercalated and oxidized V_2_C MXenes (OA-V_2_C) [38]. Based on the SEM images in Figure 1b–d and Figure S1b,c, the M-V_2_C, A-V_2_C, O-V_2_C and OA-V_2_C show a similar accordion-like layered morphology, which is beneficial to the rapid Li^+^ diffusion. TEM-EDS mapping (Figure 1e) shows that V, O, C and Na elements are evenly distributed in OA-V_2_C, demonstrating the successful pre-intercalation of Na^+^ in OA-V_2_C. XRD patterns of V_2_AlC, M-V_2_C, A-V_2_C, O-V_2_C and OA-V_2_C are displayed in Figure 2a. Compared with the diffraction peaks of V_2_AlC, the (002) peaks of M-V_2_C, A-V_2_C, O-V_2_C and OA-V_2_C moved left after the modification, and the characteristic reflection of V_2_AlC at 13.3° and 41.2° almost disappeared, revealing that the Al element was selectively etched from V_2_AlC and the ultimate formation of V_2_C MXenes. In addition, there were still some impurities such as vanadium and aluminum oxides presented after the etching process for the derived four specimens, which is similar to previous reports [39,40].

In order to investigate the chemical compositions and bond states of M-V_2_C, A-V_2_C, O-V_2_C and OA-V_2_C, XPS spectra are presented in Figure 2b–e and Figure S2. It can be found that the V, O, C and Na elements in OA-V_2_C based on the whole XPS survey (Figure 2b) are consistent with the above EDS elemental mappings. There are three pair deconvoluted peaks at 523.5/516.5, 522.6/514.9, 521.4/513.8 eV which can be ascribed to V^4+^, V^3+^ and V^2+^ in OA-V_2_C (Figure 2c) [39,40]. The appearance of V^4+^ peaks can be attributed to vanadium oxide, indicating that V_2_CT_X_ MXene is inevitably slightly oxidized during etching, as well as alkalized and oxidized treatment. The peaks at 531.4, 529.8 and 533.8 eV in the high-resolution O 1s XPS spectrum (Figure 2d) can be assigned to V-OH, V-O and H-O-H bonds, indicating the substitution of oxygen-containing groups. Through alkalized and oxidized treatment, V_2_CTX MXene substitutes -F and -OH groups with -O groups. These -O substitutions enhance pseudocapacitive properties and facilitate Na ion diffusion, resulting in more active sites available for ion storage in OA-V_2_C. Figure 2e displays the high-resolution C 1s spectrum, which can be deconvoluted into four peaks at 288.5, 286.3, 284.8 and 282.5 eV, corresponding to the C=O, C-O, C-C and C-V bonds [41]. The atomic percentages of F and O in M-V_2_C, A-V_2_C, O-V_2_C and OA-V_2_C based on the XPS results prove the reduced F surface termination and increased O-terminate groups in O-V_2_C, A-V_2_C and OA-V_2_C (Table S1). Most importantly, the OA-V_2_C occupies the highest content of oxygen, illustrating that the alkalization and oxidation co-modification could greatly reduce F surface termination and concurrently increase O-terminate groups in OA-V_2_C. The composition of the samples with a few Al certifies that highly pure V_2_C MXene was successfully obtained.

**Figure 3:**
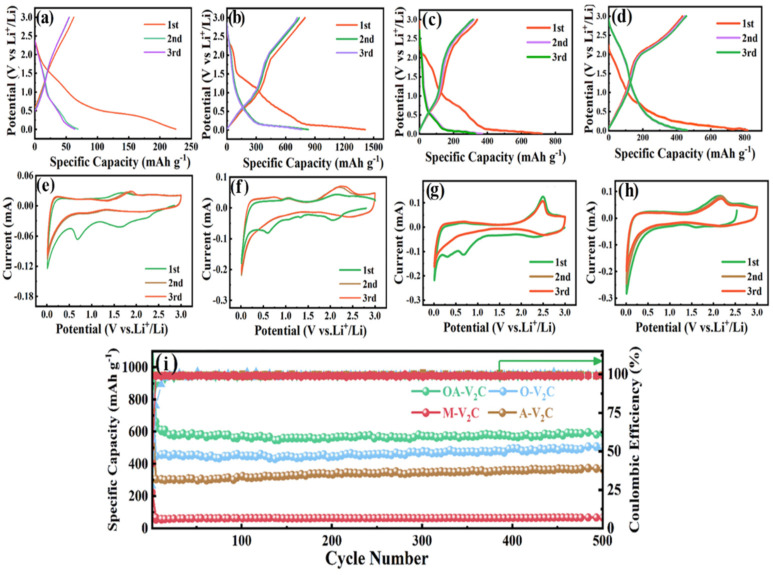
Galvanostatic charge–discharge profiles at 0.2 A g^−1^ for (**a**) M-V_2_C, (**b**) OA-V_2_C, (**c**) A-V_2_C and (**d**) O-V_2_C. The initial three CV curves of (**e**) M-V_2_C, (**f**) OA-V_2_C, (**g**) A-V_2_C and (**h**) O-V_2_C. (**i**) Cycling performance of M-V_2_C, OA-V_2_C, A-V_2_C and O-V_2_C at 0.2 A g^−1^.

The electrochemical performances of OA-V_2_C, A-V_2_C, O-V_2_C and M-V_2_C anodes were tested in LIBs (Figure 3). Figure 3a–d represent the galvanostatic charge–discharge curves of the initial three cycles for the M-V_2_C, OA-V_2_C, A-V_2_C and O-V_2_C anodes, respectively. The M-V_2_C shows the lowest charge–discharge capacity in the initial cycle with an initial Coulombic efficiency (ICE) of 27.6% at 0.2 A g^−1^, which might be attributed to the limited interlayer spacing and unsatisfied electrical conductivity of M-V_2_C. Conversely, the OA-V_2_C delivered charge–discharge capacity of 798.9/1414 mAh g^−1^ with the highest ICE of 56.5%. The A-V_2_C anode exhibited a charge–discharge capacity of 721.5/382.3 mAh g^−1^ with an ICE of 53.0%, and the O-V_2_C anode displayed a charge–discharge capacity of 820.1/443.2 mAh g^−1^ with an ICE of 54.0% The irreversible capacity loss in the first cycle for the four electrodes could be attributed to the formation of a solid electrolyte interface (SEI) layer [42,43,44]. The charge–discharge curves of the OA-V_2_C anode at various current densities in Figure S3 with low potential polarization validate the excellent electrochemical kinetics. Figure 3e–h display the CV curves of the M-V_2_C, OA-V_2_C, A-V_2_C and O-V_2_C anodes at 0.1 mV s^−1^ in the initial three cycles. In the first cathodic scan, the obvious reduction peaks weakened or even vanished for the four samples. The increase in O-terminated groups on the modified V_2_CT_x_ surface may explain why there is an enhanced ability to uptake Li^+^. In the following cycles, the CV curves nearly overlap, indicating the excellent reversibility of the modified V_2_CT_x_ electrodes [45]. Figure 3i displays the cycling performance of the four electrodes at 0.2 A g^−1^. It can be observed that the OA-V_2_C possesses a reversible capacity of 601 mAh g^−1^ underneath 500 cycles at 0.2 A g^−1^ corresponding to a Coulombic efficiency of 100%. In contrast, the A-V_2_C, O-V_2_C and M-V_2_C electrodes just delivered capacities of 389, 502 and 71 mAh g^−1^ undergoing 500 cycles. It is noted that the lithium ion storage capability of OA-V_2_C is significantly improved after alkalization and oxidation co-modification. The increased -O groups and the interlayer intercalation of Na^+^ ions enlarges the interlamellar space and increases the active sites of the material, which is convenient for more Li^+^ ion insertion in the MXene layer. The -O functional group modification could improve the Li^+^ ion storage performance undergoing the adjustment. OA-V_2_C typically have various surface terminations, such as hydroxyl (-OH), oxygen (-O) or fluorine (-F), which can significantly increase their electrochemical activity. These functional groups could supply additional sites for ion storage, leading to higher capacities. Furthermore, OA-V_2_C demonstrate pseudocapacitive behavior, where charge storage occurs not only through conventional faradaic reactions but also via surface or near-surface redox reactions. This can contribute to higher charge storage capacities by leveraging fast redox reactions at or near the electrode surface. Defects and vacancies within the MXene structure can act as additional active sites for ion accommodation or adsorption, further increasing the charge storage capacity.

**Figure 4:**
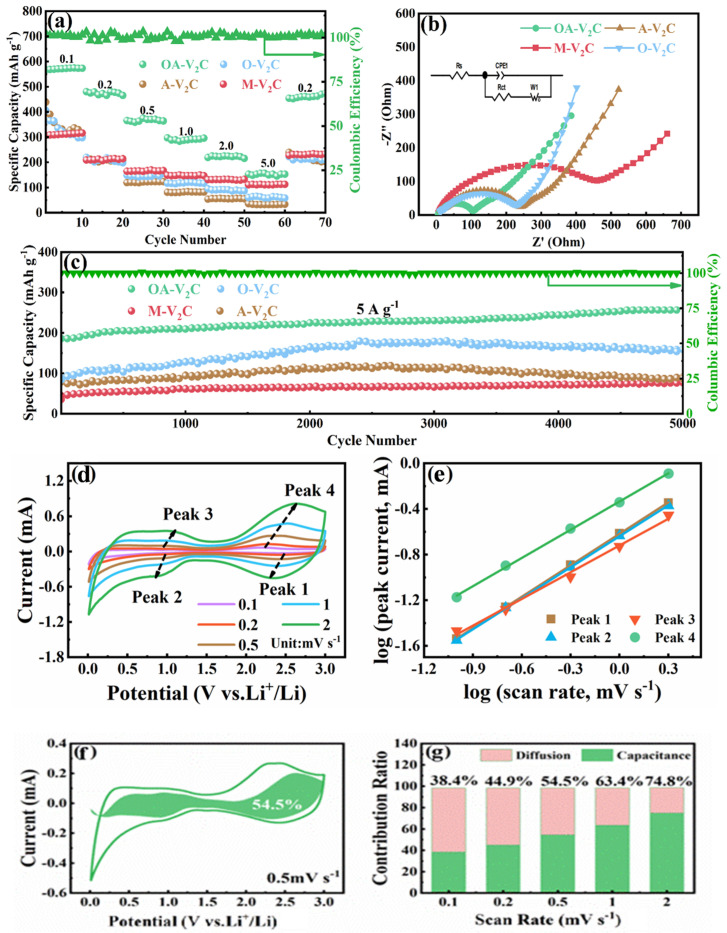
(**a**) Rate performances of M-V_2_C, OA-V_2_C, A-V_2_C and O-V_2_C. (**b**) EIS spectra of M-V_2_C, OA-V_2_C, A-V_2_C and O-V_2_C; insert image shows corresponding equivalent circuit model. (**c**) Long-term cycling performance of M-V_2_C, OA-V_2_C, A-V_2_C and O-V_2_C at 5 A g^−1^ after 5000 cycles. (**d**) CV curves of OA-V_2_C electrode at different scan rates from 0.1 to 2 mV s^−1^ and (**e**) Log(i) versus log(v) plot. (**f**) The proportion of capacitive contribution at a scan rate of 0.5 mV s^−1^. (**g**) Capacitive contribution ratios of OA-V_2_C electrode at various scan rates.

Figure 4a shows the rate performance of the four samples. The OA-V_2_C anode possesses the most competitive rate capability with discharge capacities of 567.7, 474.8, 370.2, 291.4, 222.2 and 152.8 mAh g^−1^ at current densities of 0.1, 0.2, 0.5, 1, 2 and 5 A g^−1^, respectively. As the current density returns to 0.2 A g^−1^, the reversible capacity recovers to 474.3 mAh g^−1^. In contrast, the M-V_2_C, A-V_2_C and O-V_2_C electrodes just deliver capacities of 98.3, 27.3 and 47.2 mAh g^−1^ at 5 A g^−1^. To further investigate the electrochemical behavior of OA-V_2_C anode, the electrochemical impedance spectra (EIS) of M-V_2_C, OA-V_2_C, A-V_2_C and O-V_2_C after 500 cycles are displayed in Figure 4b with the equivalent circuit model in the inset. It can be seen that the charge-transfer resistance (R_ct_) of OA-V_2_C (94.6 Ω) is the smallest among the four samples. The R_ct_ values are estimated to be 269.2, 257.4 and 463.2 Ω for the A-V_2_C, O-V_2_C and M-V_2_C samples, respectively. It is noted that the Na pre-intercalation and generated surface oxygen groups could effectively improve the electronic conductivity after the alkalization and oxidation treatment. Long-term cycling performance of the four specimens at 5 A g^−1^ for 5000 cycles is offered in Figure 4c. The OA-V_2_C electrode shows the most excellent stability with a high capacity of 252 mAh g^−1^ remained after 5000 prolonged cycles. Even after 5000 cycles, the capacity keeps increasing, while the capacity of A-V_2_C and O-V_2_C rapidly declines after 2500 cycles. The M-V_2_C anode delivered an ultralow capacity at 5 A g^−1^ which might be due to the poor electrical conduction and moderate interlayer spacing. A comparison of electrochemical performances between OA-V_2_C and various MXene-based anode materials in LIBs is displayed in Table S2, disclosing the competitive performance of the OA-V_2_C [40,45,46,47,48,49,50]. To further investigate the rapid Li^+^ storage kinetics of the OA-V_2_C electrode, CV curves at various scan rates ranging from 0.1 to 2 mV s^−1^ are recorded, as shown in Figure 4d. Typically, the relationship between the scan rate (v) and the peak current (i) can be described as follows:

*i* = a*v*^*b*^ (1)

A b value of 0.5 suggests that the capacity is governed by the ion diffusion process, whereas a b value of 1.0 indicates a capacitive-controlled process [43,44]. Subsequently, according to Equation (2), the slopes of four redox peaks are calculated to be 0.92, 0.91, 0.79 and 0.83, respectively (Figure 4e). This result shows that the Li^+^ insertion/extraction in OA-V_2_C anode can be attributed to both capacitive and diffusion behaviors. In addition, the capacitive (k_1_*v*) and diffusion (k_2_*v*^1/2^) contributions in CV curves are determined by subsequent Equation (3) [51,52,53].

Log*i* = loga + blog*v* (2)

*i* = k_1_*v*^1/2^ + k_2_*v* (3)

**Figure 5:**
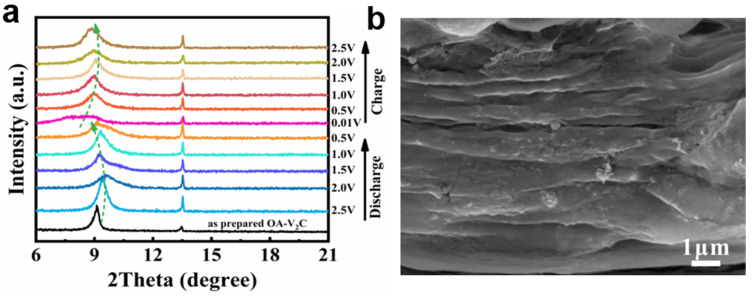
(**a**) Ex situ XRD patterns of OA-V_2_C during charge–discharge process. (**b**) SEM image of the OA-V_2_C after 5000 cycles.

The capacitive contribution is assessed to be 54.5% at 0.5 mV s^−1^ for the OA-V_2_C anode (Figure 4f). The capacitive contribution ratios at different scan rates are displayed in Figure 4g, in which the capacitive contribution ratios rise from 54.5% to 74.8% with the increase in the sweep rates. All of these results show that the capacitive charge storage plays an important role in the capacity of OA-V_2_C, which is the reason for the excellent charge-transfer kinetics [54,55,56,57]. The ex situ XRD patterns during the charge–discharge process in Figure 5a confirms the (de)intercalation mechanism of Li^+^ in the OA-V_2_C electrode. Upon discharge, the (002) peaks of OA-V_2_C shift left, caused by the increased distance of the interlayer. Conversely, the (002) peaks of OA-V_2_C shift right during the charging process, illustrating the highly reversible property of OA-V_2_C. Most importantly, the layered architecture can still be maintained even after 5000 cycles (Figure 5b)**,** illustrating the excellent structural stability of OA-V_2_C. This innovative refinement methodology paves the way for building high-performance V_2_CT_x_ MXenes anode materials in LIBs.

## 4. Conclusions

In summary, OA-V_2_C, O-V_2_C, A-V_2_C and M-V_2_C samples were prepared via a hydrothermal reaction and evaluated as anode materials in LIBs in this work. Among the four samples, the OA-V_2_C demonstrates the most excellent performance among the four specimens with a high reversible capacity (601 mAh g^−1^ at 0.2 A g^−1^ after 500 cycles), excellent rate performance (222.2 mAh g^−1^ and 152.8 mAh g^−1^ at 2 A g^−1^ and 5 A g^−1^, respectively) and stable long-term cycling performance (252 mAh g^−1^ at 5 A g^−1^ after 5000 cycles). It is noted that the oxidation modification greatly reduces the -F group and improves the reversible capacity. On the other hand, the introduction of Na^+^ into V_2_CT_x_ MXenes significantly stabilizes interlayer construction and hinders the aggregation of nanosheets, expediting charge transfer and Li^+^ diffusion efficiency. It is noted that the surface terminations, such as hydroxyl (-OH), oxygen (-O) or fluorine (-F), can significantly increase the electrochemical activity. These functional groups could supply additional sites for Li ion storage, leading to higher capacity. Furthermore, OA-V_2_C demonstrate pseudocapacitive behavior, where charge storage occurs not only through conventional faradaic reactions but also via surface or near-surface redox reactions. This can contribute to higher charge storage capacities by leveraging fast redox reactions at or near the electrode surface. Defects and vacancies within the MXene structure can act as additional active sites for ion accommodation or adsorption, further increasing the charge storage capacity. This special modification for V_2_CT_x_ MXenes in this work provides an efficient pathway for building high-performance anode materials in LIBs.

## Data Availability

The raw data supporting the conclusions of this article will be made available by the authors on request.

## Supplementary Materials

The following supporting information can be downloaded at: https://www.mdpi.com/article/10.3390/ma17143516/s1, Figure S1: SEM images of (a) V_2_AlC MAX phase, (b) O-V_2_C and (c) A-V_2_C; Figure S2: High-resolution XPS spectra of (a) V 2p, (b) O 1s and (c) C 1s for M-V_2_C. High-resolution XPS spectra of (d) V 2p, (e) O 1s and (f) C 1s for A-V_2_C. High-resolution XPS spectra of (g) V 2p, (h) O 1s and (i) C 1s for O-V_2_C; Figure S3: Charge–discharge curves of OA-V_2_C anode at different current densities; Table S1: Atomic percentages of V, O, C, F, Na and Al elements in M-V_2_C, O-V_2_C, A- V_2_C and OA-V_2_C based on XPS results; Table S2: Comparison of electrochemical performances between OA-V_2_C and various MXene-based anode materials in LIBs.
