# Supplementary material for: Tailoring Alkalized and Oxidized V2CTx as Anode Materials for High-Performance Lithium Ion Batteries
Source: Materials (Basel). 2024 Jul 16;17(14):3516. doi: 10.3390/ma17143516 (PMC11278483; doi:10.3390/ma17143516)
Supplement: Supplementary file 1 [file materials-17-03516-s001.zip › materials-3068789-supplementary.pdf]

Tailoring Alkalized and Oxidized V_2CT_x as Anode Materials for High-Performance Lithium Ion Batteries

Yuxuan Zhang[1], Lin Gao[1*], Minglei Cao[1] and Shaohui Li[2*]

[1] Hubei Key Laboratory of Energy Storage and Power Battery, School of Mathematics, Physics and Optoelectronic Engineering, Hubei University of Automotive Technology, Shiyan 442002, PR China.
[2] School of Materials Science and Engineering, Zhengzhou University, Zhengzhou 450001, China;
*Corresponding Author E-mail: gaolinctgu@hotmail.com (L.G.) and shaohuili@zzu.edu.cn (S. Li)

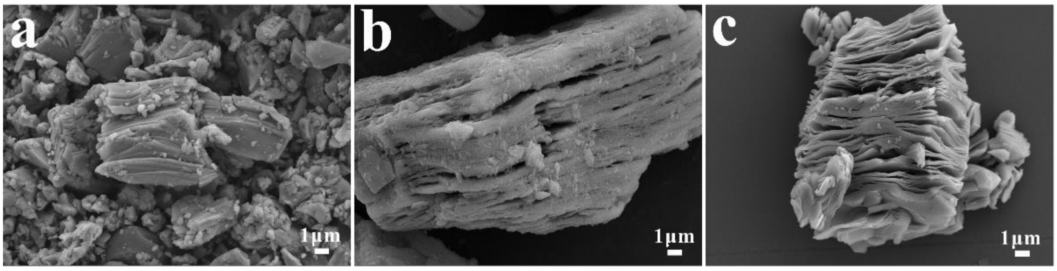

Figure S1 SEM images of (a) V_2AlC MAX phase, (b) O-V_2C and (c) A-V_2C.

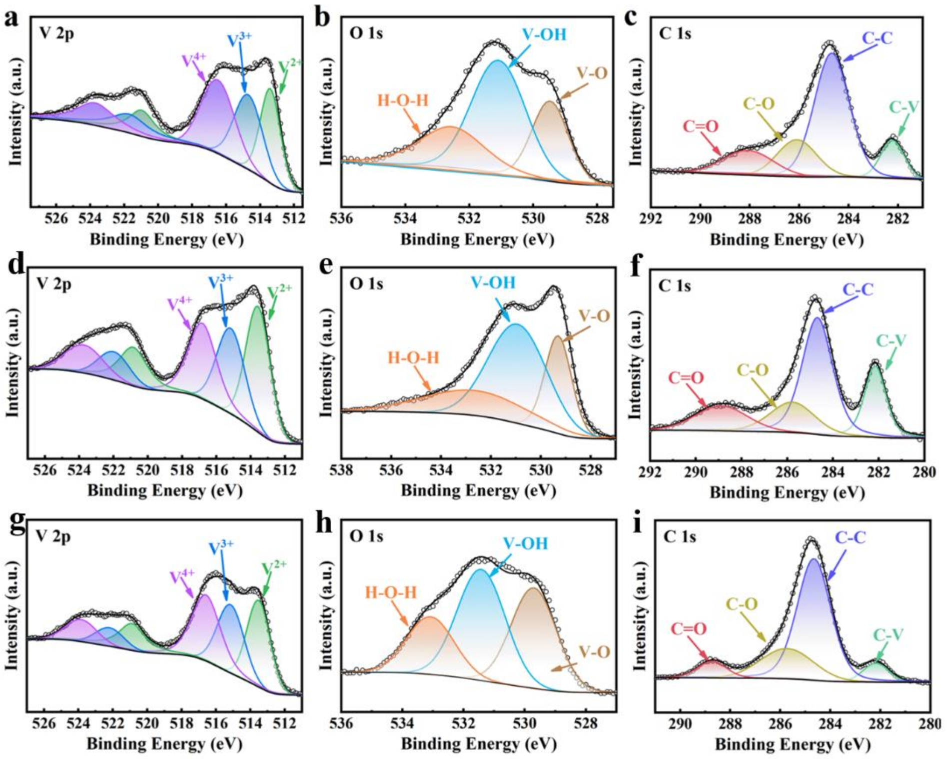

Figure S2 High resolution XPS spectra of (a) V 2p , (b) O 1s and (c) C 1s for M-V_2C. High resolution XPS spectra of (d) V 2p, (e) O 1s and (f) C 1s for A-V_2C. High resolution XPS spectra of (g) V 2p, (h) O 1s and (i) C 1s for O-V_2C.

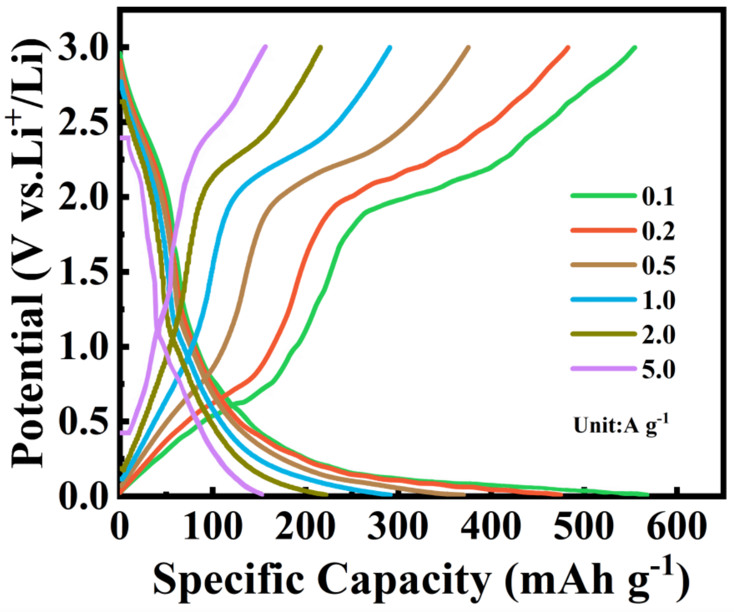

Figure S3 Charge-discharge curves of OA-V_2C anode at different current densities.

Table S1 Atomic percentages of V, O, C, F, Na and Al elements in M-V_2C, O-V_2C, A-V_2C and OA-V_2C based on XPS results.

Samples	V	O	C	F	Na	Al
M-V_2C	13.7	19.54	32.73	24.42	7.25	2.35
OA-V_2C	21.79	32.21	29.02	2.35	12.8	1.83
A-V2C	22.65	30.75	28.5	3	13.01	2.01
O-V2C	17.37	26.16	50.55	3.24	0.55	2.13

Table S2 Comparison of electrochemical performances between OA-V_2C and various MXene-based anode materials in LIBs

Sample	Current Density	Cycle number	Reversible Capacity (mA h g^{-1})	Ref.
Oxidized V_2CT_x	1 A g^{-1}	1000	125	40
Ti_3C_2	0.26 A g^{-1}	100	123.6	45
$(V_{0.7}Ti_{0.3})_2CT_x$	0.2 C	100	254	46
NaV_6O_{15}	1 A g^{-1}	200	120	47
V_2C MXene	0.5 A g^{-1}	500	243	48
VCT-K	1 A g^{-1}	2500	153.5	49
V_2CT_x-N	1 A g^{-1}	50	233	50
**OA-V_2C**	**0.2 A g^{-1}**	**500**	**601**	**This work**
	**5 A g^{-1}**	**5000**	**252**	
